# Evaluation of a dedicated brain metastases treatment planning optimization for radiosurgery: a new treatment paradigm?

**DOI:** 10.1186/s13014-016-0593-y

**Published:** 2016-02-02

**Authors:** Thierry Gevaert, Femke Steenbeke, Luca Pellegri, Benedikt Engels, Nicolas Christian, Marie-Thérèse Hoornaert, Dirk Verellen, Carine Mitine, Mark De Ridder

**Affiliations:** Department of Radiotherapy, Universitair Ziekenhuis Brussel, Vrije Universiteit Brussel, Brussels, Belgium; Department of Radiotherapy, Centre Hospitalier Jolimont, Jolimont, Belgium

**Keywords:** Stereotactic radiosurgery, Brain metastases, Automated planning optimization, Single isocenter dynamic conformal arc

## Abstract

**Purpose:**

To investigate the feasibility of a novel dedicated treatment planning solution, to automatically target multiple brain metastases with a single isocenter and multiple inversely-optimized dynamic conformal arcs (DCA), and to benchmark it against the well-established multiple isocenter DCA (MIDCA) and volumetric modulated arc therapy (VMAT) approaches.

**Material and Methods:**

Ten previously treated patients were randomly selected, each representing a variable number of lesions ranging between 1 to 8. The original MIDCA treatments were replanned with both VMAT and the novel brain metastases tool. The plans were compared by means of Paddick conformity (CI) and gradient index (GI), and the volumes receiving 10 Gy (V10) and 12 Gy (V12).

**Results:**

The brain metastases software tool generated plans with similar CI (0.65 ± 0.08) as both established treatment techniques while improving the gradient (mean GI = 3.9 ± 1.4). The normal tissue exposure in terms of V10 (48.5 ± 35.9 cc) and V12 (36.3 ± 27.1 cc) compared similarly to the MIDCA technique and surpassed VMAT plans.

**Conclusions:**

The automated brain metastases planning algorithm software is an optimization of DCA radiosurgery by increasing delivery efficiency to the level of VMAT approaches. Improving dose gradients and normal tissue sparing over VMAT, revives DCA as the paradigm for linac-based stereotactic radiosurgery of multiple brain metastases.

## Introduction

Radiation therapy has become a popular treatment option in the management of patients with brain metastases. The role and effectiveness of stereotactic radiosurgery (SRS) alone and in combination with whole brain radiation therapy (WBRT) for the treatment of brain metastases has been evaluated in two randomized clinical trials [1, 2]. One decade later, debate continues on to the role of SRS as the first line of care treatment modality for brain metastases patients, yet the controversy has shifted from overall survival to quality of life and how this relates to neurocognitive decline, which is a more appropriate endpoint in times of personalized medicine. In the absence of level-1 evidence, best practice guidelines recommend a close monitoring of patients treated solely with SRS for an early detection of distant recurrences. The guidelines of the American Society of Radiation Oncology only support SRS without concurrent WBRT for patients with up to four brain metastases [3]. The reported successful radiosurgical outcome for two patients with ten or more metastases suggested at the absence of an essential number of metastases that can be treated with radiosurgery [4, 5]. Recently, Yamamoto et al. performed a multi-institutional prospective observational trial (JLGK0901) and concluded that SRS without WBRT as the initial treatment for patients with five to ten brain metastases is non-inferior to SRS alone in those with two to four brain metastases, in terms of overall survival [6]. Although a randomized trial should confirm these results, evidence grows that SRS alone is an important treatment option for brain metastases patients, irrespective of the number of metastases.

Different radiosurgery technologies are available and accomplish the high ablative dose with a sharp dose fall-off in their respective ways. The dose distribution generated with a gantry-mounted linear accelerator (LINAC)-based SRS system is accomplished using multiple intersecting non-coplanar dynamic conformal arcs (DCA), multiple intensity-modulated beams or volumetric modulated arc therapy (VMAT). A previous study in our department showed that for linac-based SRS, DCA is still favorable to sculpt the dose around the lesion with respect to SRS treatment aims (i.e., high conformity and sharp gradient) [7]. Therefore, our standard of care for SRS treatments of multiple brain metastases is a multi isocentric set-up, aligning the micro multi-leaf collimator (MLC) around every individual lesion using DCA. This complex time consuming treatment planning translates in extended treatment delivery times, as it requires patient repositioning for every individual metastases.

Recently, a new dedicated brain metastases treatment planning solution has been developed, intended to carefully balance normal tissue protection, target coverage and treatment efficiency. The automated brain metastases treatment planning software, named Elements, generates radiosurgery-grade treatment plans to treat up to ten metastases simultaneously with a single isocenter.

The purpose of the current study was to investigate the feasibility of this novel treatment planning software for single isocenter SRS treatment in patients with multiple brain metastases and to compare the dosimetry and efficiency against the well-established multiple isocenter dynamic conformal arc (MIDCA) approach and the single isocenter VMAT approach.

## Material and methods

### Patient population

Ten patients were randomly selected from the pool of previously treated brain metastases patients with the Novalis at the UZ Brussel hospital. They represent a variable number of lesions, range of target sizes and shapes most frequently observed in the practice of SRS for brain metastases. The number of lesions varied from 1 (*n* = 1), 2 (*n* = 2), 3 (*n* = 3), 4 (*n* = 1), 7 (*n* = 2) to 8 (*n* = 1). The gross target volume (GTV) was defined as the area of contrast enhancement on MRI and was expanded with 2 mm based on our experienced and previous published data on intrafraction motion [8]. After delineation of targets and critical structures on MRI data sets, the CT data with their related DICOM Radiotherapy Structure Sets were transferred to several dedicated treatment planning systems: iPlan dose v.4.5 (Brainlab AG, Feldkirchen, Germany) for MIDCA treatment planning, Eclipse RapidArc v10 (Varian, Palo Alto, CA, USA) for VMAT treatment planning, and to the automated brain metastases treatment planning software (Brainlab Elements, Brainlab AG, Feldkirchen, Germany) for single isocenter dynamic conformal arc (SIDCA) treatment planning. The treatment dose of 20 Gy was prescribed to the 80 % isodose line covering 99.5 % of the target volume. The dose to the surrounding healthy brain and critical structures (brainstem, cochlea, optical nerve, eyes and lens) were optimized to minimize complications for all the plans generated. A Novalis Tx (Brainlab AG, Feldkirchen, Germany) radiosurgery linac with 6 MV photon energy equipped with an integrated high definition multi-leaf collimator (Varian, Palo Alto, CA, USA), consisting of 120 leaves, including 64 central leaves with a width of 2.5 mm and 56 peripheral leaves with a width of 5 mm [9], was modelled for all treatment planning purposes.

### Dedicated automated brain metastases treatment planning

**Fig. 1:**
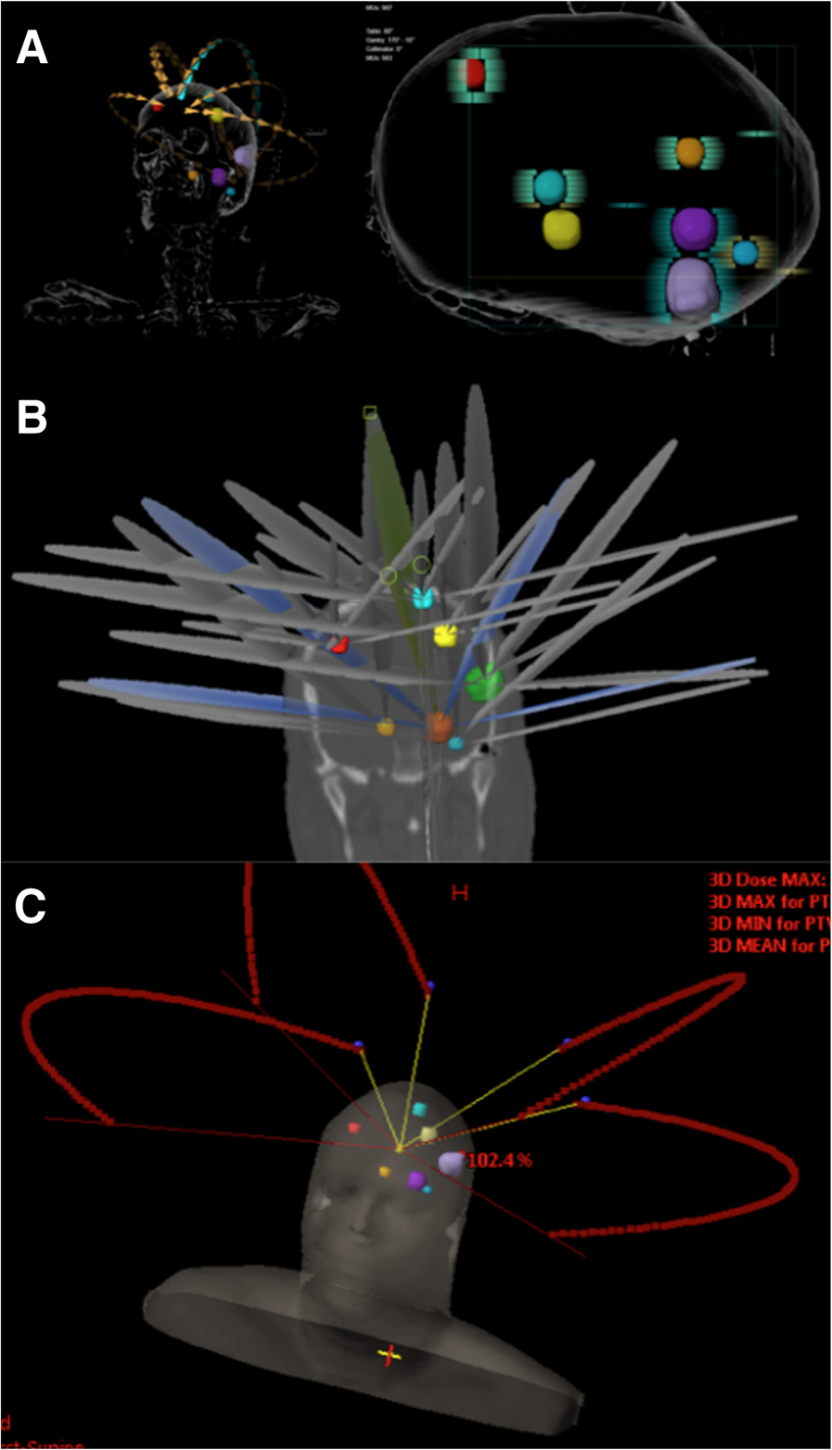
Arc arrangement for the three different treatment modalities: (**a**) Single isocenter dynamic conformal arc (SIDCA), (**b**) Multi isocenter dynamic conformal arc (MIDCA) and (**c**) Volumetric Modulated Arc technique (VMAT)

This novel automated software solution is designed to simulate treatments for multiple individual metastases simultaneously. The contours are automatically imported for a correct comparison. The isocenter position is determined as the average position of the centers of mass of each individual target. The dose prescription is defined as the dose applied to 99.5 % of the target volume and this coverage is guaranteed by the optimization algorithm. The software uses a pre-configured set of non-coplanar DCA to treat the targets. The algorithm starts by considering a maximum of five couch positions and two independent arcs per couch position. By default, the couch positions are defined so that the arcs are equally distributed and opposing arcs are avoided. Depending on which hemisphere of the head the isocenter is located in, the arcs are mirrored about the sagittal plane. The start and stop angles of the arc are first set to default values and automatically modified during optimization. The MLC leaf positions are shaped conform to the targets, with an additional margin of up to 1 mm in addition to any margin that may have been defined in the template, for all fields of each arc. To prevent irradiation of normal tissue, all targets must be assigned to specific arcs and not necessarily treated by all arcs at the same time. In the latter scenario, it might occur that multiple targets line up along the direction of motion of a leaf pair, which would imply the pair to open widely and also expose the healthy tissue in between the targets. Therefore, each leaf pair is only allowed to expose one target at any time. This decision of assigning lesions to a specific arc is automatically performed by the algorithm itself. For the sake of efficiency, the algorithm bases this choice on the principle that as many targets as possible should be treated by each arc. The choices are made so that minimal number of arcs is used based on the amount of parallel lesions that can be treated by one arc. Additionally, a large range of collimator rotations is explored to smear out the overdosage caused by the radiation leakage between MLC leaves. Although this rotation is limited because all targets treated by an arc still have to fit into the effective MLC field, its contribution to the protection of healthy tissue is still deemed to be significant. After assigning individual targets to arcs, the weights of the arcs are optimized in terms of conformity and expressed by the conformity index (CI), one for each target. In addition to optimizing the arc weights, the algorithm evaluates several other approaches to improve the conformity further, like adding or subtracting small margins around the targets with suboptimal CI and varying start and stop angles of each arc. Figure 1a shows an example of the number of arcs, arrangement used for the treatment of a patient with 7 brain metastases.

### Established radiosurgery treatment strategies

The DCA modality [10], which is used routinely at our department for the SRS treatments, is a rotational arc delivery technique in which the leaves move during the rotation of the gantry to dynamically adapt the shape of the treatment beam to the projection of the target. In this study, one isocenter for each target with 4–5 arcs equally distributed over 100–150° was used to treat the target. The couch angles of the arcs were manually selected to minimize dose spread in normal brain as well as avoiding multiple targets to line up along the direction of an arc. Forward planning is applied to shape the prescription isodose (80 %) conform to the lesions. The isocenter of each group was at the center of mass of the associated target.

The VMAT plans were developed according to the technique described by Clark et al. [11]. Constraints to targets and OAR were applied in order to achieve the planning objectives. Concentric ring-structures were utilized during optimization to achieve the steepest dose fall-off possible. Huang et al. [12] suggested VMAT plans with 4 arcs in order to achieve the steepest dose fall-off. The 4-arc plans consisted of one 358° axial arc and three 171° arcs at couch angles of 45°, 90°, and 315°. During optimization, priorities were adjusted during optimization to achieve the best results for each patient. VMAT Rapidarc (Varian, Palo Alto, CA, USA) consists of optimizing a dose distribution from dose–volume objectives. The optimizer is enabled to continuously vary the dose rate, MLC leaf positions and gantry rotation speed. The optimization itself is based on the “Progressive Resolution Optimization” algorithm. This optimization loop is based on a multi-resolution level and is ruled by means of a gradient back projection algorithm to minimize differences between current and desired doses in the cost function built on dose volume objectives defined by the planner. Variations of MLC shapes, dose rate or gantry speed settings are performed in the search of the optimal solution. An intrinsic stochastic component will avoid traps in local minima during this search.

Figure 1b and c, respectively for MIDCA and VMAT, shows the arc arrangement, length and number used in order to optimize a plan for a patient with 7 brain metastases.

### Plan comparison

In the radiosurgery community, the Paddick CI and gradient index (GI) are two well-known indices to analyze how conformal the prescription dose sculpts the target volume and how steep the dose fall-off is in the brain. The Paddick CI, providing simultaneously information about the irradiation of the target volume and healthy tissues, was used for analysis [13]. To reflect the dose fall-off outside the target, the Paddick GI was used [14].

Blonigen et al. [15] found a higher risk for symptomatic radionecrosis for plans showing a large isodose volume receiving a dose of 12 Gy and 10 Gy. The positive correlation between the volume of the low dose outside the target and radiosurgical complications confirms the importance of the intermediate doses (10 and 12 Gy) for the prediction of complications. That is why we also evaluated the volume of brain receiving a dose of 10 Gy (V10) and 12 Gy (V12), respectively. The volume receiving a low isodose (5 Gy) was also analyzed.

### Statistical analysis

A student *t*-test (Microsoft Office Excel; Microsoft Corporation, Redmond, WA, USA) was performed to obtain *p*-values to evaluate the differences between the different indices of the systems.

## Results

**Fig. 2:**
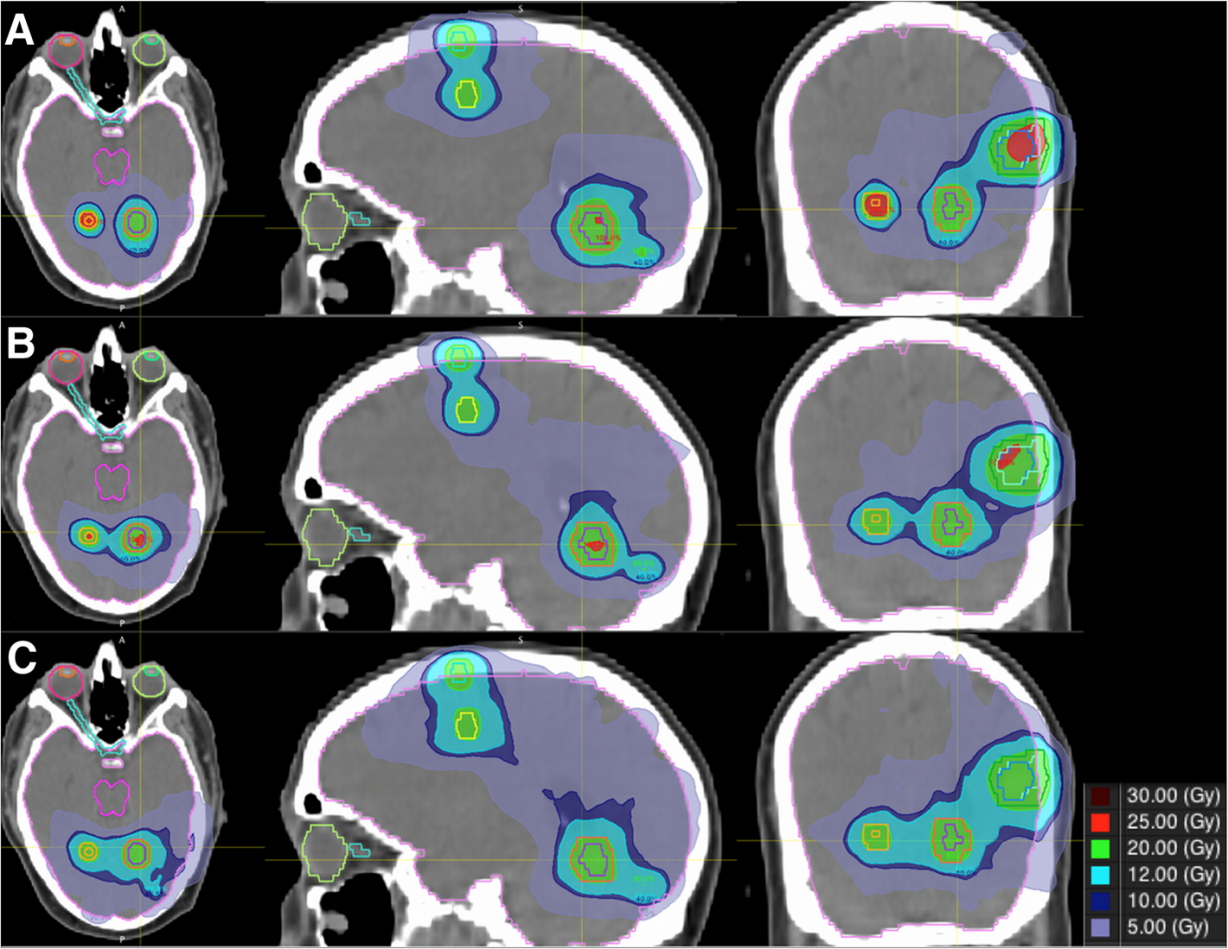
Dose distribution for a patient with 7 brain metastases for the different treatment modalities: (**a**) Single isocenter dynamic conformal arc (SIDCA), (**b**) Multi isocenter dynamic conformal arc (MIDCA) and (**c**) Volumetric Modulated Arc technique (VMAT)

Ten patients with in total 40 lesions were planned with the novel SIDCA treatment planning element, MIDCA and VMAT. The mean lesion size was 3.15 cc (range 0.14–24.61 cc). The mean total treated volume per patient was 10.60 cc (range 0.60–28.66 cc). All plans were judged clinically acceptable regarding target coverage and organs at risk sparing, although differences in dosimetrical parameters were observed. Table 1 summarizes the different indices. Figure 2 gives an example of dose distribution achieved for a patient with 7 brain metastases with the three different planning systems.

**Table 1:** Different dosimetrical paramters (i.e., conformity index (CI), gradient index (GI) and volume receiving 12Gy (V12), 10Gy (V10) and 5Gy (V5) for the different treatment techniques: multiple isocenter dynamic conformal arc (MIDCA), single isocenter DCA and volumetric modulated arc therapy (VMAT)

		CI	GI	V12 (cc)	V10 (cc)	V5 (cc)
1–3 lesions (*n* = 14)	MIDCA	0.67 ± 0.06	3.9 ± 1.5	30.4 ± 24.6	39.5 ± 31.0	131.1 ± 107.6
	SIDCA	0.67 ± 0.07	3.3 ± 0.6	31.9 ± 24.9	41.6 ± 31.0	118.9 ± 85.1
	VMAT	0.69 ± 0.20	5.3 ± 2.2	38.1 ± 28.9	52.9 ± 39.9	191.1 ± 174.1
>3 lesions (*n* = 26)	MIDCA	0.66 ± 0.08	4.9 ± 1.5	45.8 ± 32.2	68.2 ± 50.8	258.9 ± 175.8
	SIDCA	0.63 ± 0.08	4.5 ± 1.7	45.0 ± 34.8	62.3 ± 48.3	247.0 ± 219.0
	VMAT	0.65 ± 0.14	8.5 ± 3.0	62.9 ± 49.6	98.0 ± 80.6	418.0 ± 246.3
All lesions (*n* = 40)	MIDCA	0.66 ± 0.07	4.5 ± 1.6	35.6 ± 26,4	49.0 ± 38.1	173.7 ± 138.0
	SIDCA	0.65 ± 0.08	3.9 ± 1.4	36.3 ± 27.1	48.5 ± 35.9	161.6 ± 143.6
	VMAT	0.67 ± 0.16	7.1 ± 3.1	46.3 ± 35.9	67.9 ± 55.9	266.7 ± 216.7

The mean conformity of the automated single-isocenter planning tool (SIDCA plans) compared similarly to the established MIDCA and VMAT treatment techniques (CI_SIDCA_ = 0.65 ± 0.08, CI_MIDCA_ = 0.66 ± 0.07 and CI_VMAT_ = 0.67 ± 0.16). Comparable mean dose fall off was observed between SIDCA and MIDCA (GI_SIDCA_ =3.9 ± 1.4 and GI_MIDCA_ = 4.5 ± 1.6). On the other hand, compared to another single isocenter treatment, the GI and low dose of the VMAT plans (GI_VMAT_ = 7.1 ± 3.1) were significantly higher compared to the automated single isocenter planning tool (SIDCA). The V10 and V12 were significantly higher for VMAT plans (V10_VMAT_ = 67.9 ± 55.9 cc, V12_VMAT_ = 46.3 ± 35.9 cc) (*p* < 0.05) compared to MIDCA (V10_MIDCA_ = 49.0 ± 38.1 cc, V12_MIDCA_ = 35.6 ± 26.4 cc) and SIDCA (V10 = 48.5 ± 35.9 cc, V12 = 36.3 ± 27.1 cc). Low dose spread, expressed as the volume receiving 5 Gy (V5), was significantly higher for VMAT plans (V5_VMAT_ = 266.7 ± 216.7 cc) compared to the MIDCA (V5_MIDCA_ = 173.7 ± 138.0 cc) and SIDCA (V5 = 161.6 ± 143.6 cc).

If we stratify the total patient population into two groups, one comprising patients with up to 3 lesions and one comprising patients with more than 3 lesions, we found comparable conformity across both groups regardless of the delivery technique. For the gradient and low dose spread, the differences between the SIDCA and MIDCA versus VMAT were even more pronounced for patients with more than three lesions (Table 1).

## Discussion

Increasing evidence demonstrates that SRS provides highly effective and predictable local tumor control for single and multiple brain metastases. Avoidance of WBRT can have a positive impact on the patient’s cognitive status and health-related quality of life. Nowadays, there is clinical evidence to treat multiple brain metastases up to 10 upfront with a single radiosurgery session and apply repeat SRS when new lesions are detected [1–6]. Different dedicated radiosurgery systems (i.e., Gamma Knife, Cyberknife and linac-based systems) are available to perform these very precise single fraction SRS treatments. We previously compared these different devices for vestibular schwannomas and arteriovenous malformations and found that although all systems achieve SRS requirements, multiple focal entries (i.e., Gamma Knife and Cyberknife) achieve better conformity while modulated beams (Cyberknife and IMRT beams) will spread more low dose into the brain [7]. The DCA technique, which is routinely applied in our department, achieved good conformity with a reduction in low dose spread compared to the modulated beams. These results also apply to single brain metastases radiosurgery. However, for multiple brain metastases, careful selection of arc orientation is needed in order to avoid possible overlap of arcs, which will result in decreased conformity, and higher volume of low doses into the brain leading to undesirable side effects. Valéry et al. [16] determined that the risk of radionecrosis after radiosurgery is related to the presence of normal tissue included in the prescription volume and found that the conformity of a treatment plan was the only parameter influencing the risk of radionecrosis. Furthermore, dose fall-off outside the target is of equal importance as conformity. Analysis of risk factors for brain necrosis showed that V10 and V12 were the most important independent predictors of both symptomatic and asymptomatic radionecrosis indicating that volume is more important than the number of lesions treated [17]. Furthermore, Xue et al. [18] found that mean normal brain dose correlated with the total volume of the lesions rather than with the treated number of lesions.

Multiple isocenter DCA radiosurgery is less efficient in treating multiple lesions as treatment planning involves different isocenters, each with an arc arrangement. In order to reduce low dose spread, arc length and direction must be optimized. Patients also need to be repositioned for every lesion separately, which is more time consuming.

Single isocenter treatments are more efficient for multiple brain metastases. Mayo et al. [19] showed the possibility to use VMAT to treat multiple metastatic lesions using a single isocenter with multiple arcs and anticipated that the ability to treat multiple lesions simultaneously will have a significant impact on care patterns for this patient population. Although this technique looks interesting, Huang et al. [20] found an increased low-dose spread that may be of concern. This technique is designed for speed, as there is no difference in treatment time whether you treat one or multiple metastases. However, this approach comes at the cost of more radiation exposure to normal tissue, which goes against the core principles of SRS. Furthermore, based on the dose interplay effect analysis, Ma et al. [21] observed that multiple isocenter delivery (i.e., Gamma Knife and DCA) achieves better normal brain tissue sparing as compared to single isocentric or non-isocentric (i.e., CyberKnife) approaches. These conclusions are in according with our previous publication where modulated beams were found to smear the dose out over the normal tissue.

In this manuscript we propose an automated algorithm to treat single or multiple brain metastases using a single isocenter with a pre-configured set of non-coplanar DCA. All established techniques mentioned above rely on user-defined empirical values for gantry angles, collimator and couch rotations, virtual objects and their corresponding constraints. The large number of parameters and many degrees of freedom involved with traditional radiosurgery plan optimization challenges the task at hand and may result in a sub-optimal treatment plan and variable plan quality depending on the experience of the user. Despite all the parametric freedom routinely available, the planner is faced with the often conflicting ambitions of shaping the radiation dose as conformal as possible, while simultaneously optimizing the dose gradient. In this scenario, as an analogy, the jockey becomes more important than the horse. That is why it would be beneficial to have a treatment planning system automatically balancing the large number of parameters and degrees of freedom available and presenting a solution with minimal user interaction. The planning efforts for a forward planning technique such as MIDCA, scale with the number of lesions. That is why VMAT radiosurgery was likely to replace MIDCA for multiple lesions in terms of efficiency, at the cost of increased low dose spread.

The automated Elements software is also an inverse planning optimization technique that uses a single isocenter and several non-coplanar DCA, which makes for an interesting tool in terms of planning and treatment efficiency. This novel software tool generated plans with similar conformity compared to the MIDCA and VMAT. For the low dose spread, comparable results were found as for MIDCA but with a significant decrease of low dose spread compared to the VMAT plans, improving stereotactic treatment planning in terms of gradient index and low dose spread while maintaining conformity.

An important remark is that when multiple targets are separated far from each other, some of the lesions may be covered by the 5 mm MLC instead of the 2.5 mm. That is why for these lesions, if conformity seems not acceptable, a second group for that particular lesion must be created in order to achieve the planning constraints.

Furhter investigations must now be performed in order to ensure that planning reproducibility can be performed once the patient will be treated. A small angular setup uncertainty can lead to significant dosimetric degradation especially for lesions located distant from the treatment isocenter. That is why stereoscopic x-ray images and 6° of freedom positioning will be used in order to cope with those uncertainties. Previously work in our department has showed that a threshold of 1 mm and 0.5° is sufficient to assure good dose deposition [8]. In order to maintain the setup accuracy during table rotations, we suggest to perform snapshot verifications and reposition if necessary at that particular table angle [22].

## Conclusions

The automated brain metastases treatment planning element, based on an inversely-optimized SIDCA approach, revealed comparable results to the general accepted and clinical used MIDCA approach. By reducing the time on planning, patient setup and re-positioning of the treatment couch, this software tool significantly improves the treatment planning and delivery efficiency while preserving the plan quality of the MIDCA technique and lowering the low dose spread of the single isocenter VMAT approach, suggesting that this novel software offers the best of both worlds (i.e., efficient single-isocenter DCA delivery).
